# Comorbid Normal Pressure Hydrocephalus with Parkinsonism: A Clinical Challenge and Call for Awareness

**DOI:** 10.1155/2018/2513474

**Published:** 2018-01-21

**Authors:** A. Cucca, M. C. Biagioni, K. Sharma, J. Golomb, R. M. Gilbert, A. Di Rocco, J. E. Fleisher

**Affiliations:** ^1^Department of Neurology, The Marlene & Paolo Fresco Institute for Parkinson's & Movement Disorders, New York University School of Medicine, NYU Langone Medical Center, New York, NY, USA; ^2^Department of Neurosurgery, Adult Hydrocephalus Program, NYU School of Medicine, New York, NY, USA; ^3^Department of Neurological Sciences, Rush University Medical Center, Chicago, IL, USA

## Abstract

Idiopathic normal pressure hydrocephalus (iNPH) is the most common cause of hydrocephalus in adults. The diagnosis may be challenging, requiring collaborative efforts between different specialists. According to the International Society for Hydrocephalus and Cerebrospinal Fluid Disorders, iNPH should be considered in the differential of any unexplained gait failure with insidious onset. Recognizing iNPH can be even more difficult in the presence of comorbid neurologic disorders. Among these, idiopathic Parkinson's disease (PD) is one of the major neurologic causes of gait dysfunction in the elderly. Both conditions have their peak prevalence between the 6th and the 7th decade. Importantly, postural instability and gait dysfunction are core clinical features in both iNPH and PD. Therefore, diagnosing iNPH where diagnostic criteria of PD have been met represents an additional clinical challenge. Here, we report a patient with parkinsonism initially consistent with PD who subsequently displayed rapidly progressive postural instability and gait dysfunction leading to the diagnosis of concomitant iNPH. In the following sections, we will review the clinical features of iNPH, as well as the overlapping and discriminating features when degenerative parkinsonism is in the differential diagnosis. Understanding and recognizing the potential for concomitant disease are critical when treating both conditions.

## 1. Background

Idiopathic normal pressure hydrocephalus (iNPH) is a neurological condition clinically characterized by the triad of gait dysfunction, cognitive abnormalities, and urinary disorders [1]. iNPH is morphologically characterized by the expansion of the lateral and third cerebral ventricles in the absence of macroscopic obstructions to the CSF outflow. The pathogenesis of iNPH remains unknown; however CSF dynamics show two major abnormalities: a pulsatile increase in CSF pressure and an increased outflow resistance. Ventricular enlargement with near normalization of hydrostatic pressure takes place but the pathophysiology involved in these phenomena remains highly disputed [2]. Typically, iNPH shows an insidious onset without any specific prior event detectable on medical history. The progression is usually slow, spanning months or years. Gait dysfunction is usually the first reported abnormality and is characterized by reduced step height during the swing phase, reduced stride length and reduced velocity [3–5]. Equilibrium-related gait variables are particularly affected, including wide base, increased external rotation of the knees, and enlarged foot rotation angles [6]. Other frequent features include propulsion or retropulsion, festination, freezing of gait (FoG), and hesitancy of gait which may confer a “magnetic” character. Kinematic abnormalities of the lower limbs show a bilateral and symmetric distribution [7]. Dynamic balance during transitional movements, like standing or turning, is severely impaired. Cognitive symptoms of iNPH include impaired attention, short-term recall, and executive function that ultimately evolve into dementia [8]. In a recent observational study, up to 46% of patients with NPH were found to display depressive symptoms, and apathy is also common [9]. Bladder symptoms usually involve detrusor hyperactivity, manifesting as increased urinary urgency and frequency [10, 11]. According to the International Society for Hydrocephalus and Cerebrospinal Fluid Disorders guidelines, “probable NPH” is defined by the combination of gait dysfunction with insidious onset after age 40 without precipitating events, lasting at least 3–6 months in the absence of comorbidities which could fully explain the symptoms [12]. In clinical practice, the latter carries the most controversy. First, the age of these patients, usually between the 6th and the 7th decade of life, is associated with a high risk of comorbid factors potentially affecting walking, such as peripheral neuropathy, arthritis, or degenerative spine disease. Therefore it can be difficult to know whether the gait disorder is purely due to iNPH. For the same reason, a coexisting neurodegenerative condition like idiopathic Parkinson's disease (PD) or other parkinsonism must also be considered. Since each condition requires different treatments, recognizing coexisting PD and iNPH is essential.

## 2. Case Report

A 79-year-old right-handed female with lumbar spondylosis and chronic low back pain presented with a two-year, slowly progressive history of mild left hand resting tremor, stooped posture, generalized slowness, and fatigue. She reported a decline in her balance, though denied falls, and she was able to walk independently. She denied urinary symptoms, constipation, dysphagia, hypophonia, hyposmia, and symptoms of REM behavioral disorder. She denied cognitive or behavioral problems and denied previous exposure to neuroleptics, antiemetics, or head trauma. Her family history was noncontributory. Baseline examination revealed a pleasant woman with normal mental status who scored a 28/30 on the Montreal Cognitive Assessment (MoCA), with points missed for delayed recall. She displayed mild hypomimia with decreased blink rate. Myerson's sign was present, with no palmomental and no grasp reflex. Vertical saccades were slightly hypometric in both directions. There was a near-constant, low-frequency chin tremor. Her slow pill-rolling tremor of the left hand was unmasked by distraction and contralateral activation, and associated cogwheeling was appreciated in the ipsilateral wrist. There was slight rigidity affecting the left extremities and mild bilateral bradykinesia. Chair mobility was normal, and her gait notable for bilaterally diminished arm swing, affecting the left more than the right. Cadence, stride length, and ground clearance were slightly reduced. She had no FoG. She recovered from the pull test in 4 steps, yielding a UPDRS motor score of 21. The remainder of her physical examination was notable for bilateral, symmetrically reduced deep tendon reflexes in the lower limbs and a positive Romberg test. Electromyography and nerve conduction studies demonstrated a primarily sensory, large fiber peripheral neuropathy affecting both legs, with no evidence of radiculopathy, plexopathy, or myopathy. The patient was diagnosed with PD according to the United Kingdom Parkinson's Disease Society Brain Bank (UKPDSBB) criteria. She was started on rasagiline 1 mg once a day.

**Figure 1:**
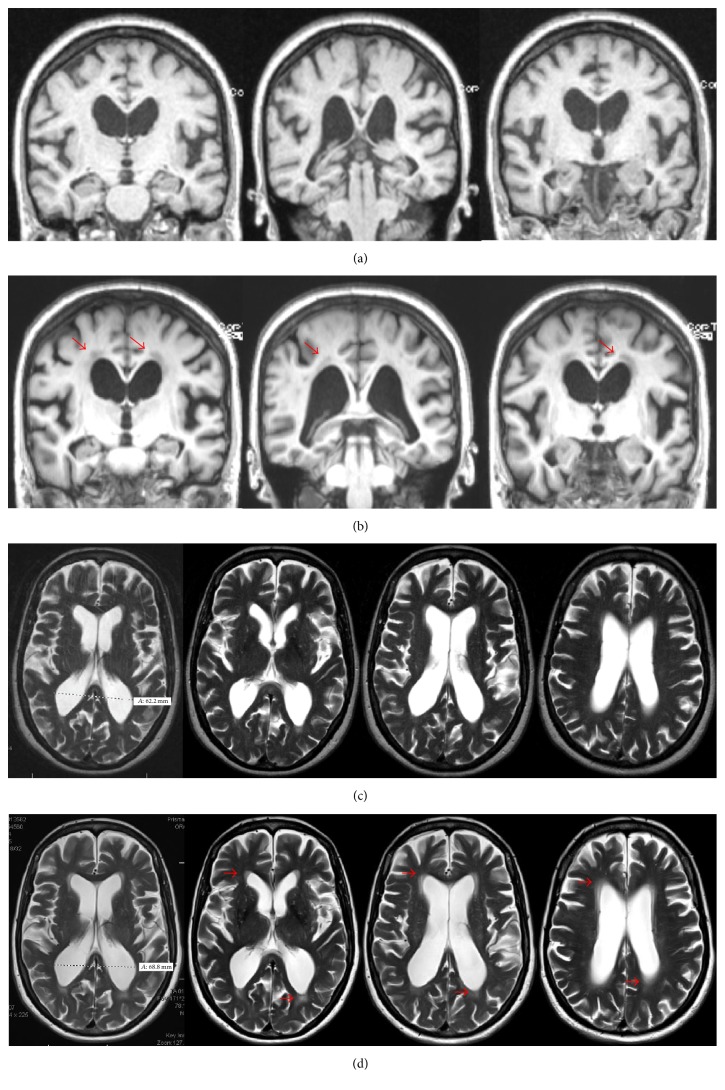
Brain MRI imaging. (a) Baseline coronal MPR sequences. (b) One-year follow-up coronal MPR sequences: periventricular white matter hypointensity (red arrows). Slight narrowing of cortical gyri on the vertex with enlarged Sylvian fissure. (c) Baseline Axial T2-weighted sequences. (d) One-year follow-up axial T2-weighted sequences: increased periventricular white matter hyperintensity (red arrows).

Six months later, she reported occasional detailed visual hallucinations with retained insight and progressive decline in her hand dexterity. Her physical examination was unchanged. A brain MRI revealed mild prominence of the ventricular system and cortical sulci along with multiple foci of periventricular, subcortical, and brainstem T2/FLAIR hyperintensities, consistent with chronic nonspecific microvascular changes (Figure 1). Rasagiline was discontinued due to a lack of efficacy and the potential contribution to hallucinations. Carbidopa-levodopa 25–100 mg was initiated and gradually titrated up to one full tablet TID. After 6 months, the patient reported improvements in stiffness, general slowness, and tremor. Her UPDRS motor score on levodopa improved by 19% and hallucinations completely resolved.

Over the following months, the patient experienced progressive gait deterioration with marked unsteadiness, particularly during transfers, with frequent falls and severe FoG. Near-falls began occurring daily, she was unable to walk unassisted when outdoors, and FoG episodes became disabling. She also reported increased urinary frequency, occasional urinary incontinence, mild forgetfulness affecting her short-term memory, and difficulties with word retrieval. Her mood significantly deteriorated, with increased apathy and pervasive sadness. On examination, her MoCA decreased to 22/30, with abnormal verbal recall, visuospatial function, executive function, and mental calculation. Her examination was notable for severe gait failure, with markedly decreased stride length and cadence, en bloc turns, freezing, and absent postural reflexes. Her gait failure was notably out of proportion to her otherwise stable appendicular signs. Carbidopa-levodopa was increased up to 1.5 tablets TID without benefit.

**Figure 2:**
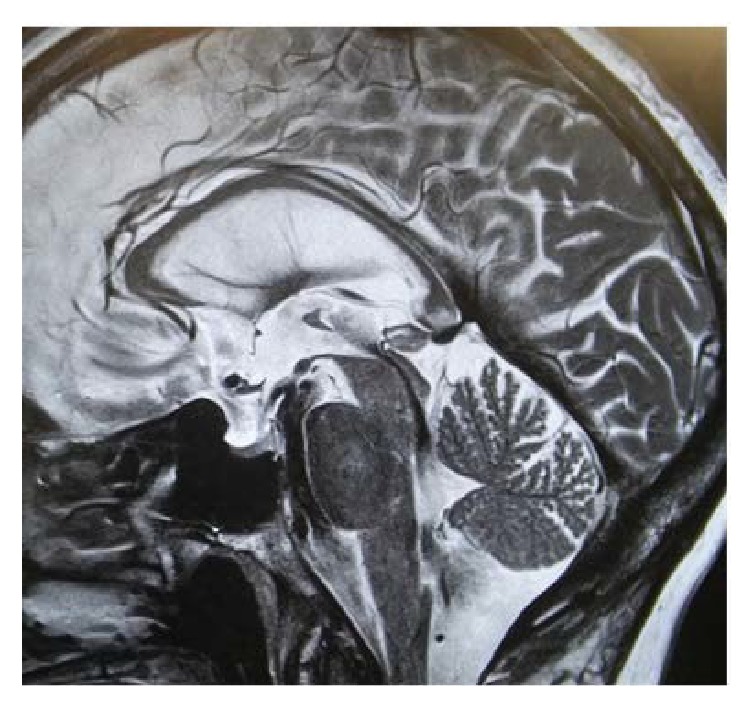
One-year follow-up brain MRI, sagittal T2 section. A strong flow void artifact is noted at the Sylvian aqueduct.

**Figure 3:**
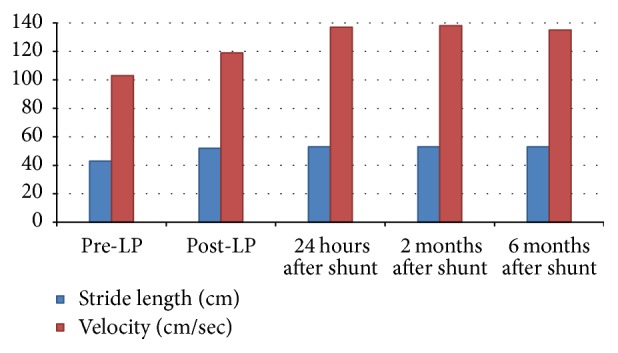
Video gait analysis at distance of 18 m.

In considering the possibility of iNPH, we ordered a repeat brain MRI (Figure 1). The ventricular size was further increased and ventriculomegaly was out of proportion to sulcal enlargement. Confluent areas of T2 hyperintensity were detected in the periventricular white matter. On sagittal sections, a strong flow void was noted at the cerebral aqueduct (Figure 2). The patient underwent a large volume lumbar puncture (LP) with pre- and post-LP testing. Approximately 40 cc of clear CSF was withdrawn. Standard CSF analysis was unremarkable. Observation of gait after LP revealed improved velocity and uniformity of cadence relative to pre-LP baseline. There was a distinct improvement in the patient's ability to make 180° turns with no evidence of FoG. Video gait analysis was performed on a distance of 18 m with postprocessing of gait speed and stride length. Both parameters significantly improved compared to her pre-LP condition (Figure 3). Additionally, her post-LP neuropsychological assessment revealed significant improvement in Trail Making B (Table 1).

Given her response to LP, a ventricular-peritoneal (VP) shunt was placed without complications. Over the following weeks, she experienced a dramatic improvement in balance and walking. She was able to walk unassisted for long distances. Her functional independence, per the Schwab and England Activities of Daily Living scale, improved from 80% (doing most of daily chores takes twice as long) to 100% (completely independent, able to do all chores without slowness, difficulty, or impairment). Falls, near-falls, and her bladder symptoms ceased. UPDRS part II FoG item improved from 2 to 0. On examination, walking speed, stride length, and heel strike significantly increased, with resolution of FoG. Her pull test normalized. Gait profile and neuropsychological assessment were repeated according to the same baseline procedures approximately two and six months after shunt placement (Figure 3 and Table 1). On both postshunt assessments, video analysis showed a significant and sustained improvement in stride length and mean gait velocity. Neuropsychological assessment revealed a significant improvement in all measured outcomes, and her MoCA improved by 3 points. Improvements in gait and balance were sustained over one year of follow-up. Appendicular symptoms including bradykinesia, stiffness, and resting tremor persisted but were well controlled without adjustments in the patient's dopaminergic regimen.

## 3. Discussion

Our patient presented with a two-year history of progressive slowness, fatigue, and mild asymmetric resting tremor. According to UKPDSBB, the clinical diagnosis of PD requires the presence of bradykinesia and at least one of the following: muscular rigidity, 4–6 Hz resting tremor, and postural instability. Baseline physical examination revealed the presence of all cardinal features included in the UKPDSBB criteria for PD, and our patient was diagnosed accordingly. In 2015, the International Movement Disorder Society (MDS) published revised PD diagnostic criteria [15]. The presence of “recurrent falls because of impaired balance within 3 years of onset” is among the red flags arguing against a diagnosis of clinically established PD. Notably, each red flag must be countered by at least one supportive diagnostic criterion, including a clear and dramatic response to dopaminergic therapy (a ≥30% improvement on clinical examination, for which our patient's 19% initial improvement did not qualify), unequivocal motor fluctuations, levodopa-induced dyskinesias, rest tremor of a limb, and either olfactory loss or cardiac sympathetic denervation. In our case, while she initially met the UKPDSBB criteria for PD, the patient's rapid progression of gait impairment and recurrent falls within 3 years are red flags arguing against idiopathic PD; her rest tremor constitutes one of the supportive criteria to argue* for* the PD diagnosis; however two supportive criteria are required to counterbalance these red flags. The concomitant presence of NPH may challenge the practical applicability of these criteria in the setting of comorbidities. Alternatively, this discrepancy may suggest that she has parkinsonism, but not idiopathic PD. It is likely that a longer follow-up period will provide meaningful observational data to either confirm or challenge the diagnosis of underlying PD.

Approximately 2.5 years after her PD diagnosis, our patient's gait and balance dramatically deteriorated, a far more rapid decline than expected for PD and disproportionate to her otherwise well-controlled parkinsonian symptoms. Optimization of dopaminergic therapy is considered the first line strategy to relieve FoG and improve gait function in PD; however this was ineffective [16]. Additionally, she reported the insidious onset of psychological, cognitive, and urologic symptoms. For all of the above reasons, we considered the possibility of NPH. From a clinical viewpoint, gait dysfunction is mandatory to diagnose iNPH. At least one more abnormality, namely, urinary symptoms or cognitive impairment, is required to support a probable diagnosis [17]. Radiologically, iNPH is characterized by a noncongenital, nonobstructive enlargement of ventricular size not attributable to cerebral atrophy, as indicated by an Evans index greater than 0.3. Supportive criteria include enlargement of the temporal horns of the lateral ventricles out of proportion to the degree of hippocampal atrophy; upbowing of the corpus callosum with callosal angle ≥ 40°; and abnormal MRI signal intensity of the periventricular white matter [18]. Additionally, the Sylvian fissure is typically enlarged, particularly when compared to otherwise tight cortical sulci with crowding of the gyri at the vertex (the so-called “high convexity sign”). Long echo-time brain MRI sequences can detect the aqueduct flow void artifact due to hyperdynamic CSF flow. Our patient's MRIs were obtained at baseline and approximately one year later when iNPH was suspected. Although slice differences limit optimal comparability, a subtle high convexity sign was appreciable on her follow-up MRI, as was moderate ventriculomegaly, periventricular white matter changes, and a prominent flow void at the Sylvian aqueduct; however the imaging did not demonstrate disproportionately enlarged subarachnoid spaces or a grossly enlarged Sylvian fissure. Our patient underwent a large volume tap test, yielding a marked objective improvement in gait function. She was diagnosed with comorbid iNPH and successfully treated with VP shunt. Dramatic benefits in gait, neuropsychological domains, and clinical assessments were immediately observed and sustained over one-year follow-up.

Between 46 and 63% of patients with iNPH show some degree of benefit from large volume CSF subtraction [18, 19]. CSF tap test is regarded as an important test for the prediction of shunt effectiveness, with a sensitivity ranging from 70% to 80%, according to different observations [20, 21]. In the absence of a clear response to single tap test, a three day-long continuous lumbar drain can be considered to increase diagnostic accuracy. However, according to the international guidelines, a decision exclusively based on predictive tests exposes patients to a high risk of misdiagnosis. False negatives include those patients who, despite underlying iNPH, fail to significantly improve after the test, possibly due to a high degree of comorbidity or to irreversible progression of the disease following an extensive delay in diagnosis. False positives, conversely, may involve a placebo response inherent to any invasive medical procedure, particularly where the patient has expectations of benefit [22]. Hence, a positive response to a tap test should be regarded as a mere supportive criterion [23]. Shunt placement may still be considered in the absence of a clear response to predictive tests when a strong clinical suspicion remains, though the patient must be counseled appropriately and have clear expectations. CSF drainage is the only effective treatment and is generally configured between a lateral cerebral ventricle and the abdominal cavity (VP shunt). The guidelines of the American Academy of Neurology support the use of a shunt as effective therapy emphasizing that diagnostic delay is the major cause of poor therapeutic outcome [24].

## 4. Comorbid iNPH and Degenerative Parkinsonism: A Call for Awareness

The role of comorbidity in patients with iNPH must be considered according to International Society for Hydrocephalus and Cerebrospinal Fluid Disorder [25]. Identifying comorbidities in patients with iNPH is critical, allowing for optimization of the patient's care for each diagnosis, maximizing their clinical outcome. As recently observed by Broggi et al., given current demographic trends, the coexistence of iNPH and neurodegenerative diseases is expected to increase [26]. According to a Swedish population study, the prevalence of iNPH is approximately 200/100,000 in the range of age from 70 to 79 [13]. According to preliminary data from an ongoing prospective single-center study, approximately 45% of patients meeting the diagnostic criteria of probable iNPH show concurrent signs of parkinsonism [27]. Importantly, these patients display decreased striatal uptake on I123-ioflupane (DatScan) single-photon emission computed tomography (SPECT), consistent with an underlying degenerative parkinsonism. Recently, Odagiri et al. retrospectively analyzed 127 patients with definite iNPH [14]. Twenty-one of these patients reported parkinsonian symptoms including tremor, hypomimia, and stiffness and were referred for a metaiodobenzylguanidine SPECT to rule out an underlying alpha-synucleinopathy. One-third of this sample showed reduced cardiac uptake. Since iNPH does not cause cardiac sympathetic-adrenergic denervation, SPECT positivity has been interpreted as an actual comorbidity between iNPH and alpha-synucleinopathy. The possibility that cooccurrence of iNPH and PD may represent more than just a comorbidity but rather a discrete phenotype resulting from a common pathogenic substrate remains to be conclusively addressed.

Diagnosing both conditions in the same patient requires a willingness to critically review the established diagnosis, appreciate deviations from the usual course, and consider potential explanations. For example, an individual with levodopa-responsive parkinsonism who develops new, pervasive gait failure and subacute cognitive complaints might have concomitant iNPH. Conversely, a patient successfully shunted for iNPH who develops progressive, asymmetric parkinsonism should raise suspicion for degenerative parkinsonism. In Table 2, we compare the main clinical and epidemiological aspects of iNPH and PD to guide the diagnostic process and highlight key differences between these two conditions. Both PD and iNPH are considered among the most common gait disorders in elderly and both are “hypokinetic movement disorders,” characterized by reduced speed, reduced stride length, and poor ground clearance along with an abnormal dynamic equilibrium manifesting as insufficient adaptation to sudden perturbations with increased risk of falling [28–30]. FoG is frequently encountered in both disorders, defined as the episodic inability to generate effective stepping manifesting with the patient's feeling of having his feet glued to the floor [31]. Generally, most clinicians tend to favor the diagnosis of iNPH in the presence of a wide-based, prominently magnetic gait with relatively spared arm swing [32]. Conversely, PD is more likely to be considered when global bradykinesia, narrow gait base, reduced arm swing, responsiveness to cues, asymmetric rigidity, bradykinesia, resting tremor, or camptocormia are observed. However, most of the behavioral and cognitive abnormalities in PD—such as executive dysfunction, apathy, depression, and bradyphrenia—can overlap with those observed in patients with iNPH [33]. Parkinsonian signs—including bradykinesia and rigidity—are commonly observed in the lower limbs of patients with iNPH. It is also a common clinical experience that mixed hydrocephalic and parkinsonian gait features coexist, with various degrees of levodopa responsiveness.

In a recent review of 16 studies including 1256 iNPH patients, Espay et al. showed that shunt responsiveness, one of the cornerstones of iNPH diagnosis, declines dramatically over longer observational periods, challenging the current diagnostic construct [34]. In these patients, the frequent finding of associated neurodegenerative pathologies on postmortem examinations suggests that ventricular enlargement may signal subclinical parenchymal changes, therefore representing the only macroscopic sign of a neurodegenerative disorder. In the absence of known secondary causes of hydrocephalus, iNPH should therefore represent a diagnosis of exclusion and the possibility of an underlying neurodegenerative disease manifesting with hydrocephalic presentation must be carefully considered. For these patients, any interventional approach should be extensively discussed and proper counseling offered, highlighting the potential for short-lived benefits from shunting.

## 5. Conclusion

Here we reported a case of comorbid iNPH in a patient suffering from idiopathic PD. Both PD and iNPH represent prevalent, treatable conditions in the elderly. Despite the recent implementation of international guidelines, diagnostic accuracy of iNPH remains poor. Without appropriate recognition of iNPH symptoms, patients may be missing the possibility of improving their quality of life and functional independence. Suspecting iNPH in the setting of PD represents an additional diagnostic challenge and may reflect a radiographic and symptomatic expression of the underlying neurodegenerative disease, though further study is required. We hope to raise awareness about the potential overlap of these two conditions to optimize patient care.

## Figures and Tables

**Table 1:** Functional independence, neuropsychological assessment, and freezing of gait.

	Pre-LP	Post-LP	Postshunt	6 months after shunt
Schwab and England ADL	80%, completely independent in most chores; takes twice as long	80%,completely independent in most chores; takes twice as long	100%, completely independent; able to do all chores without slowness, difficulty, or impairment	100%, completely independent; able to do all chores without slowness, difficulty, or impairment

MoCA	22		25	26

Trail Making A	52.0 sec	36.4 sec	29.1 sec	39.1 sec

Trail Making B	93.3 sec	95.8 sec	71.8 sec	69.7 sec

UPDRS part II FoG	2 (occasional freezing when walking)		0 (none)	0 (none)

**Table 2:** Comparing signs and symptoms of iNPH versus PD.

	iNPH	PD
Prevalence	2 cases/1000 in individuals > 70 years old [13]	10 cases/1,000 in individuals 70–79 years old [14]

Age at onset	Adults over the age of 60	Incidence peak between 70 and 79 years

Urinary disturbance	Common, nonspecific	Common, nonspecific

Cognitive dysfunction	Frontal, executive dysfunction	Frontal, executive dysfunction; global cognitive impairment usually denotes disease progression

Bradykinesia	62% of patients display bradykinesia affecting their lower limbs symmetrically; upper limbs are typically spared	Cardinal feature of the disease; upper limbs are usually affected early on in an asymmetric fashion

Rest tremor	Absent	Cardinal feature of the disease; observed in about 60% of patients

Rigidity	Rare	Cardinal feature of the disease; observed in about 60% of patients

Hallucinations	Absent	Usually manifesting with visual misperceptions and passage illusions with retained insight; florid hallucinations typically arise in advanced stages

Cortical deficits (aphasia, apraxia, agnosia)	Absent	Rare

Response to L-dopa	Absent, mild, or inconsistent	Excellent, sustained, supportive diagnostic criteria

Response to shunt placement	>60% of patients	Absent

MRI/CT	Ventriculomegaly	Noncontributory

Time course of gait failure	Early feature	If present early, regarded as a “red flag” for the diagnosis of disease

Gait velocity	Decreased	Decreased

Step length	Decreased	Decreased

Arm swing	Normal	Reduced or abolished

Freezing of gait	Early feature	Most commonly observed in the advanced stages

Responsiveness to cues	Absent or poor	Significant

Step height	Reduced	Reduced

Base	Wide	Narrow
